# Supplementary material for: Relationship between cardiac cycle and the timing of actions during action execution and observation
Source: Cognition. 2021 Dec;217:104907. doi: 10.1016/j.cognition.2021.104907 (PMC8748943; doi:10.1016/j.cognition.2021.104907)

**Supplementary Materials**

Data collected in wave one and two were pooled for statistical analysis, however we show here that comparable results were observed in both groups when analyzed separately.

*Time domain analysis*

Wave 1: The repeated measures ANOVA with factors Condition (execution and observation) and Time (0, 200, 400 and 600) revealed a significant main effect of Time (*F*(2.11, 21.11) = 10.11, *p* = .001) and no significant effects of condition or interaction between the two factors (*F*(1,10) = 1.31, *p* = .28; *F*(2.19, 21.85) = 0.24, *p* = .808) respectively.

Wave 2: The repeated measures ANOVA with factors Condition (execution and observation) and Time (0, 200, 400 and 600) revealed a significant main effect of Time (*F*(1.95, 25.30) = 13.28, *p* < .001) and no significant effects of condition or interaction between the two factors (*F*(1,13) = 0.26, *p* = .62; *F*(2.43, 31.63) = .53, *p* = .63) respectively.

*Phase domain analysis*

Supplementary Figure 1: Here we show the t-statistic at each phase for the three populations: Wave 1 (green), Wave 2 (red) and All subjects (blue). Panel A shows the data for execution and Panel B for Observation. The * show phases where the effects were significant. As is clear both groups show a qualitatively similar pattern of modulation with phase.


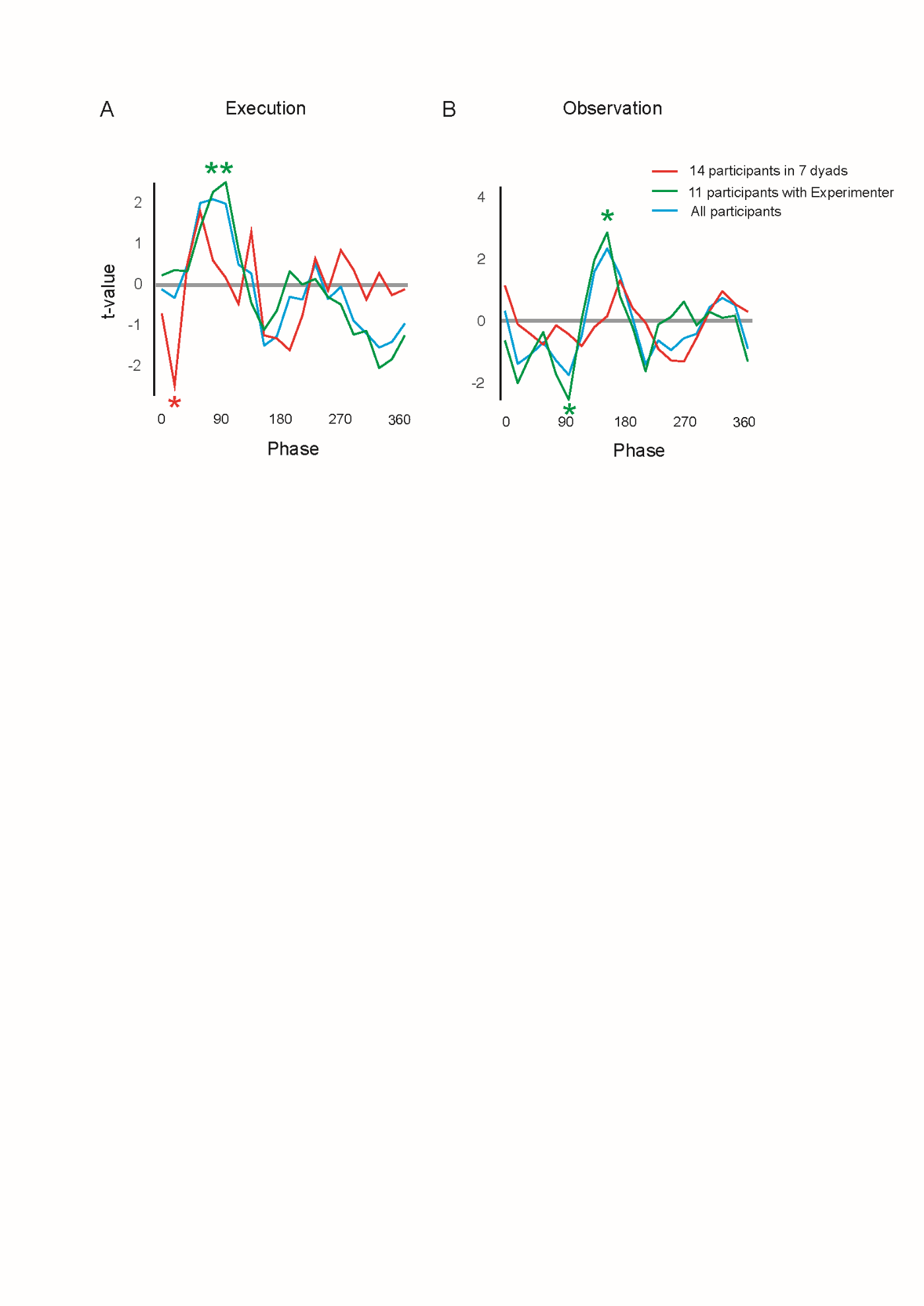

Supplement: Supplementary file 1 — Supplementary material [file mmc1.docx]
